# Comparative evaluation of conventional and modified frontalis muscle flap advancement techniques in the treatment of severe congenital ptosis: A retrospective cohort study

**DOI:** 10.1371/journal.pone.0246183

**Published:** 2021-02-02

**Authors:** Lei Zhang, Wenjuan Zhai, Lihong Yang, Chunhua Sun, Ye Pan, Hong Zhao

**Affiliations:** 1 Tianjin Eye Hospital, Tianjin, China; 2 Tianjin Key Lab of Ophthalmology and Visual Science, Tianjin, China; 3 Nankai University Affiliated Eye Hospital, Tianjin, China; 4 Clinical College of Ophthalmology Tianjin Medical University, Tianjin, China; 5 Tianjin Eye Institute, Tianjin, China; University of Toronto, CANADA

## Abstract

**Purpose:**

To introduce a modified frontalis muscle (FM) flap for use in FM flap advancement surgery and compare it with the conventional flap for correcting severe congenital ptosis.

**Methods:**

This retrospective cohort study included 200 patients (278 eyes) with severe congenital ptosis treated with FM flap advancement at Tianjin Eye Hospital from April 2018 to October 2019. The patients were divided into two groups: 100 patients (138 eyes) in the conventional group and 100 patients (140 eyes) in the modified group. The success and complication rates were evaluated.

**Results:**

The final success rate was 77.5% (107/138) in the conventional group and 90.0% (126/140) in the modified group (p = 0.005). Undercorrection was observed in 31 eyes (22.5%) in the conventional group and 14 eyes (10%) in the modified group (p = 0.005). In the conventional group, angular deformity of the upper eyelid was observed in 29 eyes (21.0%), FM paralysis in 11 (8.0%), frontal hypoesthesia in 10 (7.2%), severe hematoma in 12 (8.7%), and exposure keratitis in 8 (5.8%); these complications were not observed in the modified group (p <0.001, p <0.001, p = 0.004, p <0.001, p = 0.011, respectively). There were no cases of overcorrection, entropion or ectropion in either group.

**Conclusion:**

Compared with the conventional FM flap, the modified FM flap in this study yielded a higher success rate with a clear field of vision, mild trauma, and few complications. This technique is simple and convenient for correcting severe congenital ptosis.

## Introduction

Ptosis is one of the most common diseases in ophthalmic plastic surgery. Severe congenital ptosis not only affects the appearance of the eye but also influences visual development. In developed countries such as those in Europe as well as the United States, brow suspension is most commonly used to correct severe congenital ptosis [1], while the lack of synthetic sling materials or high import prices greatly affects the development and implementation of this surgery in other countries, especially in developing countries [2]. Frontalis muscle (FM) flap advancement is the most common procedure used to correct severe congenital ptosis in China [3]. However, this kind of operation has not been widely utilized in Europe and the United States because of its large latent separation range, which can lead to more bleeding and greater trauma than with the use of brow suspension and severe complications such as FM paralysis and frontal hypoesthesia [4–6].

The purpose of this study was to report a modified FM flap advancement approach that minimizes the shortcomings of conventional FM flap advancement surgery and to evaluate the effectiveness and safety of this modified method by comparing the success and complication rates of conventional and modified FM flap advancement surgery.

## Materials and methods

### Materials

#### Ethics statement

This retrospective cohort study (surgery on humans) was approved by the Medical Ethics Committees of Tianjin Eye Hospital and followed the Declaration of Helsinki. Due to the retrospective nature of the study, the requirement for informed consent from a parent or guardian was waived by the Medical Ethics Committees of Tianjin Eye Hospital.

A retrospective medical record review of all patients who underwent FM flap advancement surgery for upper eyelid ptosis between April 2018 and October 2019 was performed. Patients were examined 1 day, 1 week, and 1, 3, 6, 12 and 18 months after the operation. The data that were retrieved included age, sex, diagnosis, preoperative and postoperative digital photographs, margin reflex distance (MRD), lagophthalmos, and related complications, such as angular deformity of the upper eyelid, FM paralysis, frontal hypoesthesia, severe hematoma, exposure keratitis, entropion, ectropion, etc.

*Inclusion criteria*. ① conventional or modified FM flap advancement surgery completed between April 2018 and October 2019; ② patients with severe congenital ptosis (degree of ptosis ≥4 mm or MRD_1_<1 mm) and poor levator palpebrae superioris muscle function (≤4 mm). ③ follow-up period no less than 6 months.

*Exclusion criteria*. ① No Bell's Phenomena, dry eyes, FM paralysis or recurrent ptosis (noninvasive tear break-up time, tear meniscus height and meibomian gland morphology were evaluated using Oculus keratography-comprehensive analysis of the ocular surface (Oculus company, Germany) for patients with suspected dry eye.); ② inability to complete follow-up; and ③ incomplete patient data.

*If the following three criteria were met at the same time*, *the operation was considered successful*. ① a postoperative margin-to-reflex distance 1 (MRD1) between 2 mm and 4.5 mm; ② a postoperative eyelid height asymmetry no greater than 1 mm; and ③ satisfactory eyelid contour [7]. We defined undercorrections as a postoperative MRD1 less than 2 mm or an eyelid height asymmetry greater than 1 mm. We defined overcorrection as an MRD1 greater than 5 mm [8].

### Surgical technique

General anesthesia was needed for young children who were unable to undergo surgery under local anesthesia, and light intravenous sedation and local anesthesia were used in older children.

Both the conventional and modified surgeries were performed by one skin crease incision. The width of the double eyelid line incision was designed to be less than the width of the tarsal plate. After skin incision, the portion of the orbicularis oculi that covers the upper third of the tarsal plate was removed, and then the orbicularis oculi and orbital septum were separated in the direction of the eyebrow direction up to just below the supraorbital margin. The orbital septum was opened while the eyeball was gently pressed to remove the proper amount of extruding preaponeurotic fat. The FM flap was separated from the orbital part of the orbicularis muscle by subcutaneous separation at the lower edge of the eyebrow.

**Fig 1:**
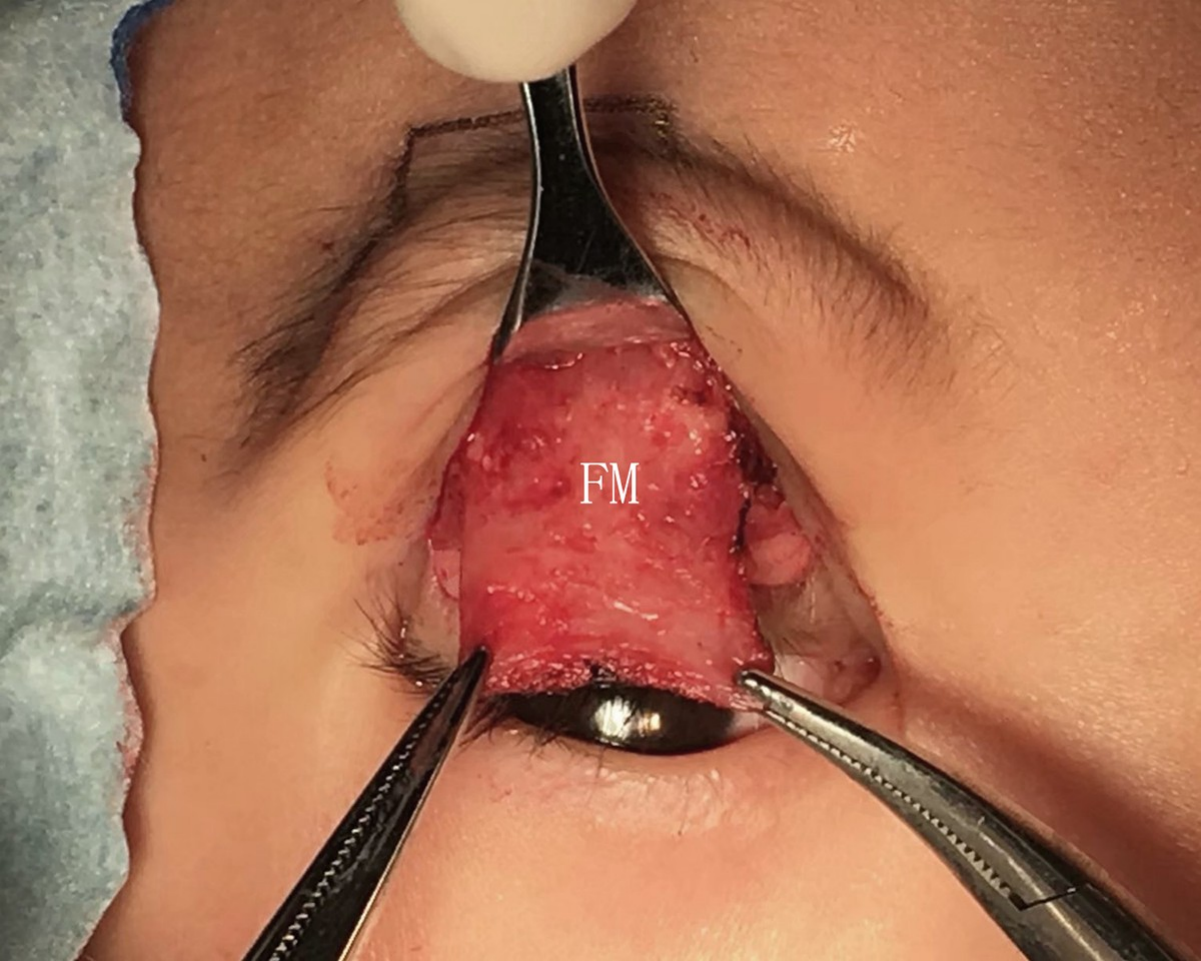
The conventional FM flap.

**Fig 2:**
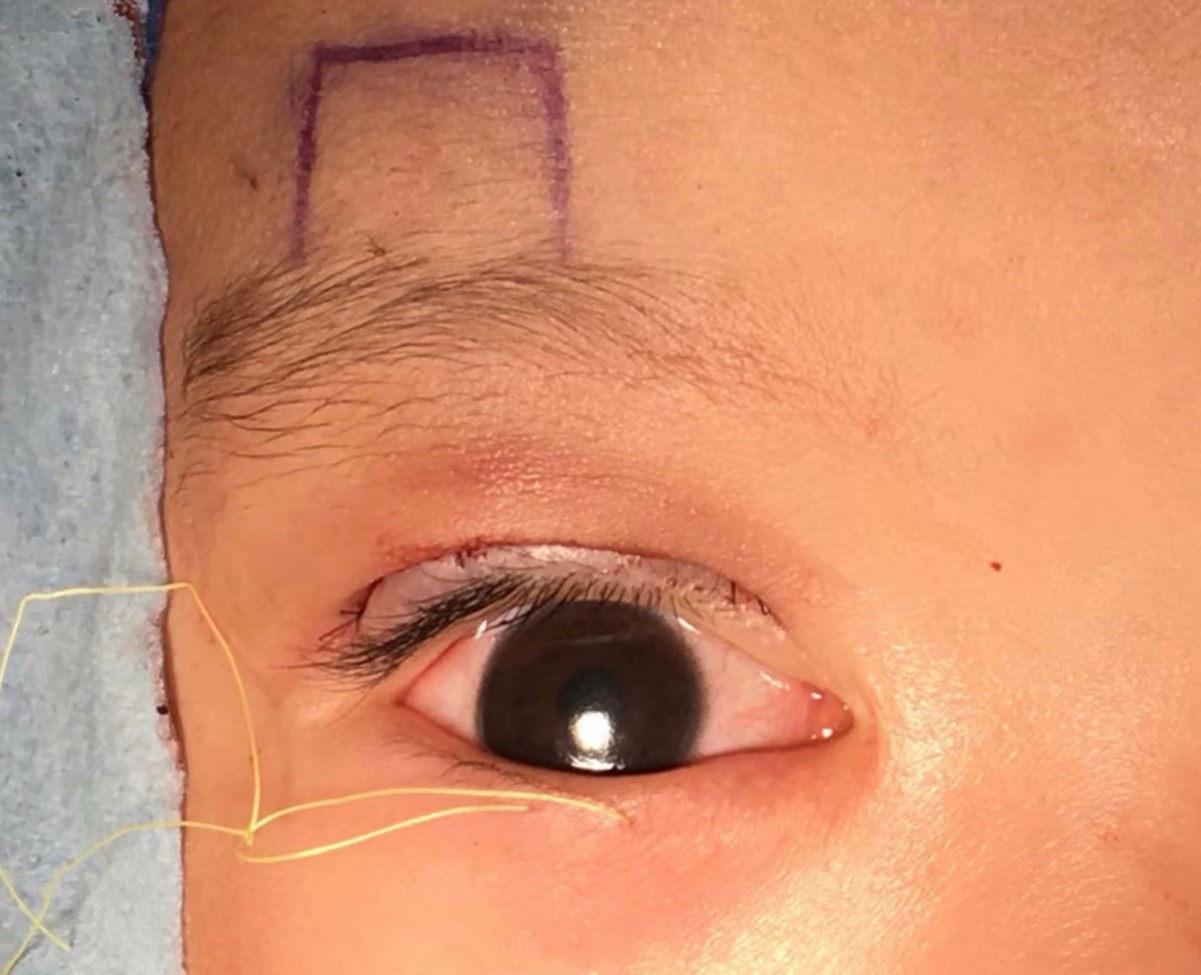
Dissection boundary of the conventional FM flap (purple line).

**Fig 3:**
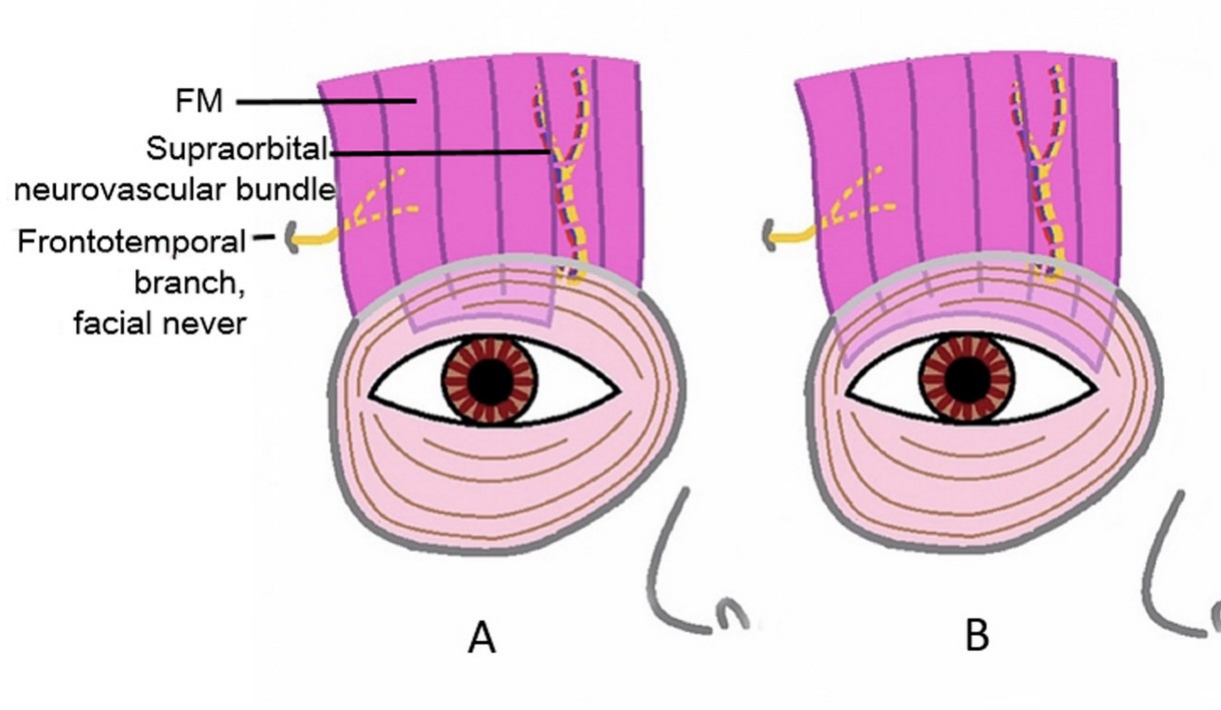
Schematic diagram showing the advancement of the FM flap to the tarsal plate. (A) The conventional FM flap; (B) The modified FM flap.

**Fig 4:**
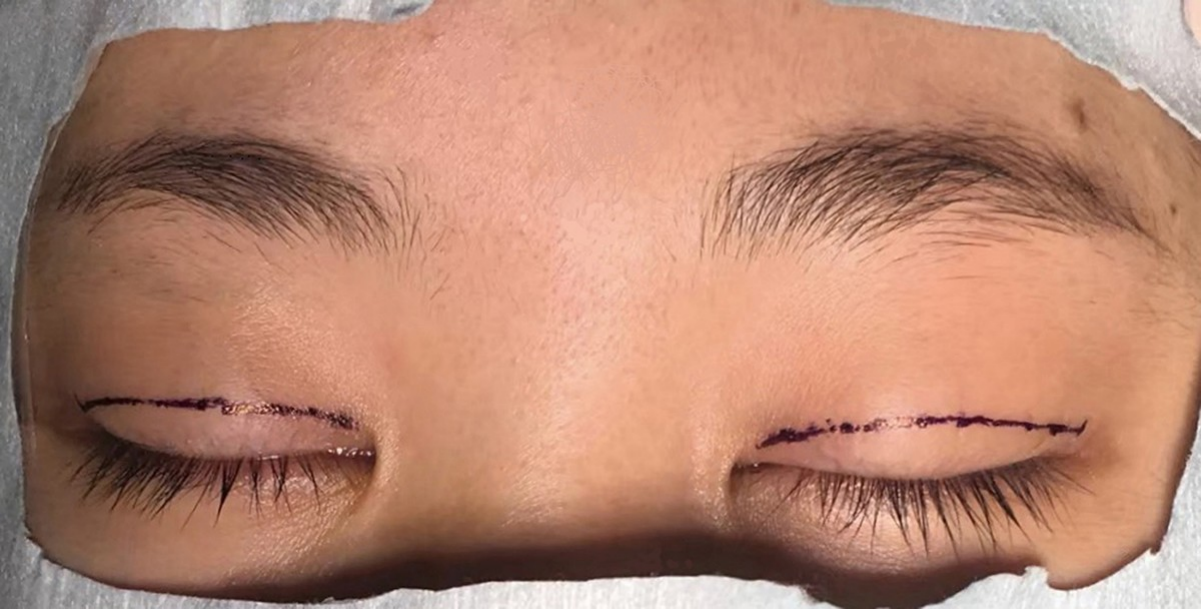
Eyelid incisions marked for the modified surgery.

**Fig 5:**
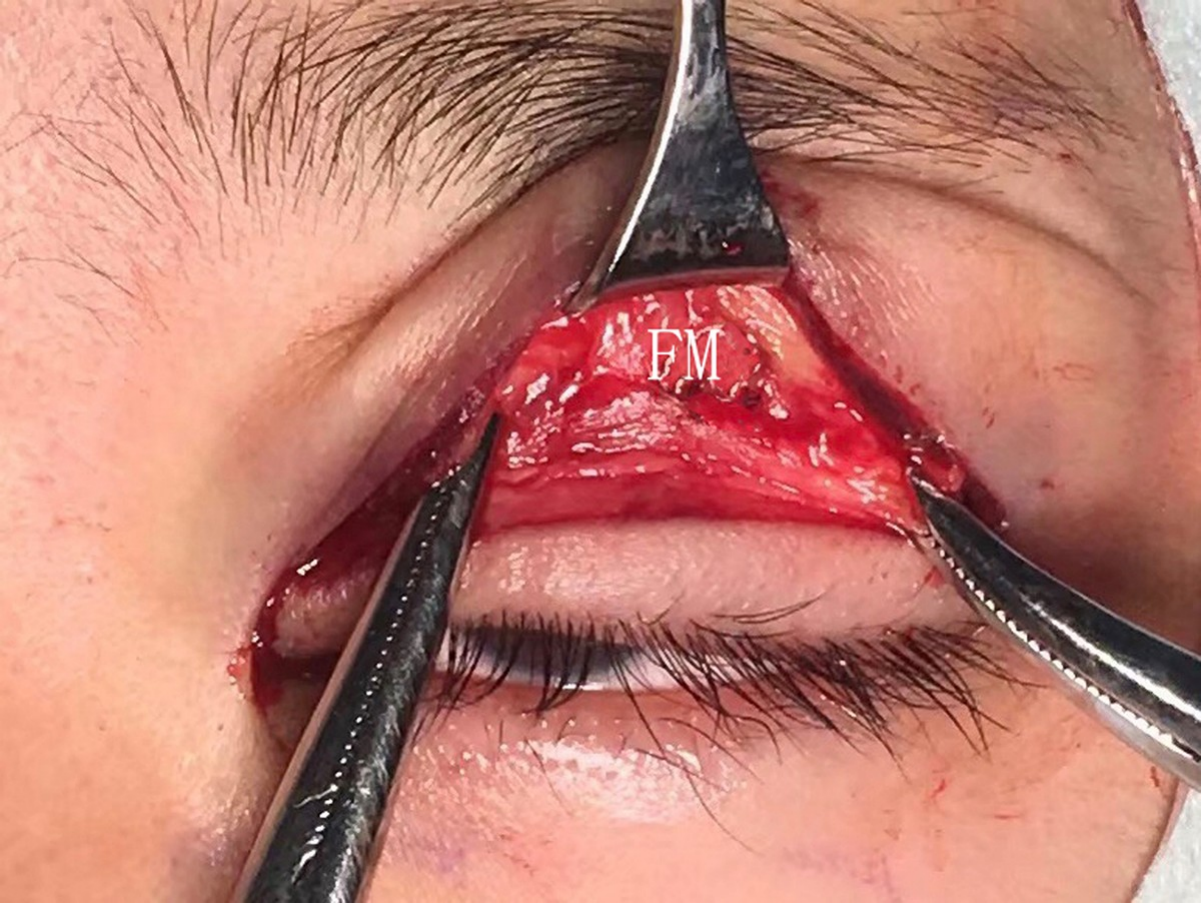
Modified FM separated from subcutaneous tissue at the lower edge of the eyebrow.

**Fig 6:**
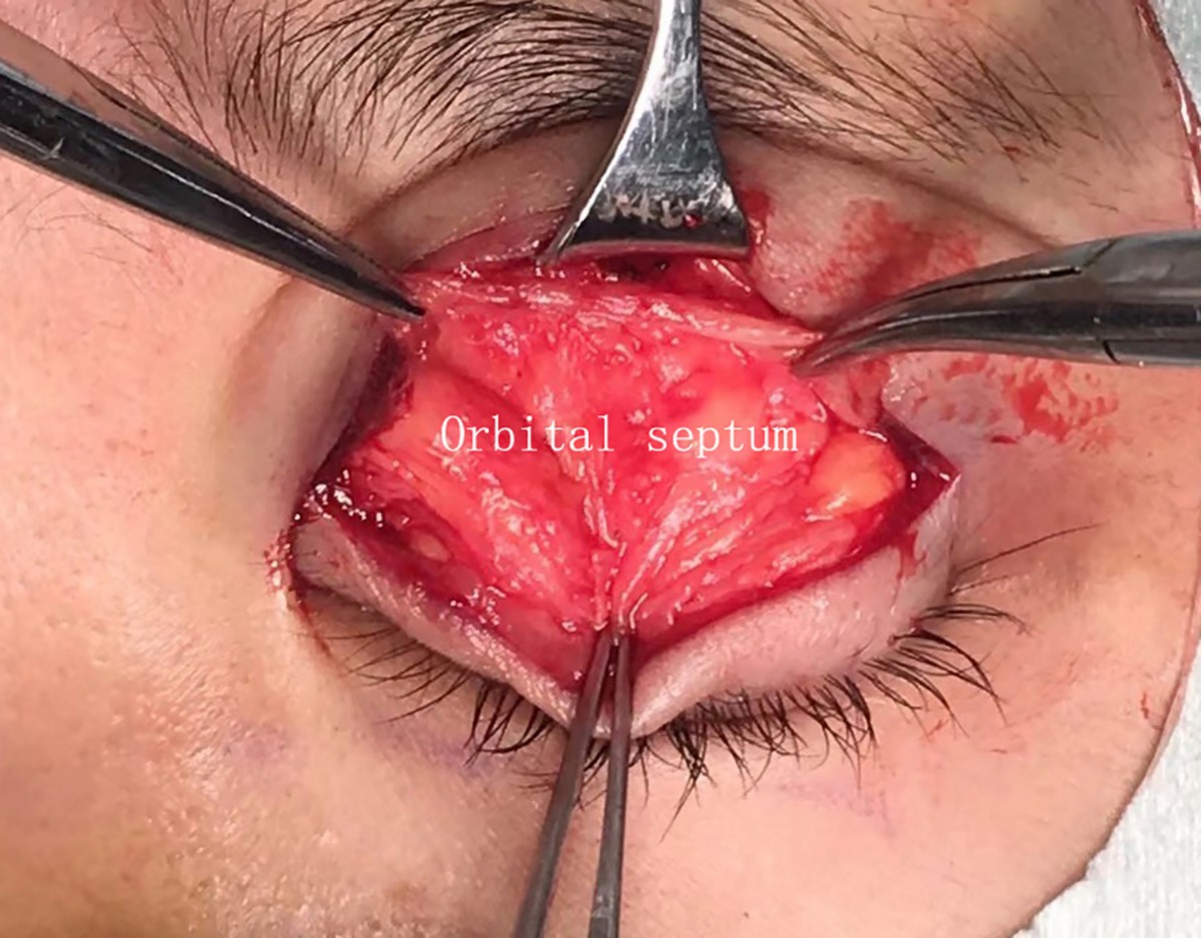
Modified FM separated from its deep surfaces.

**Fig 7:**
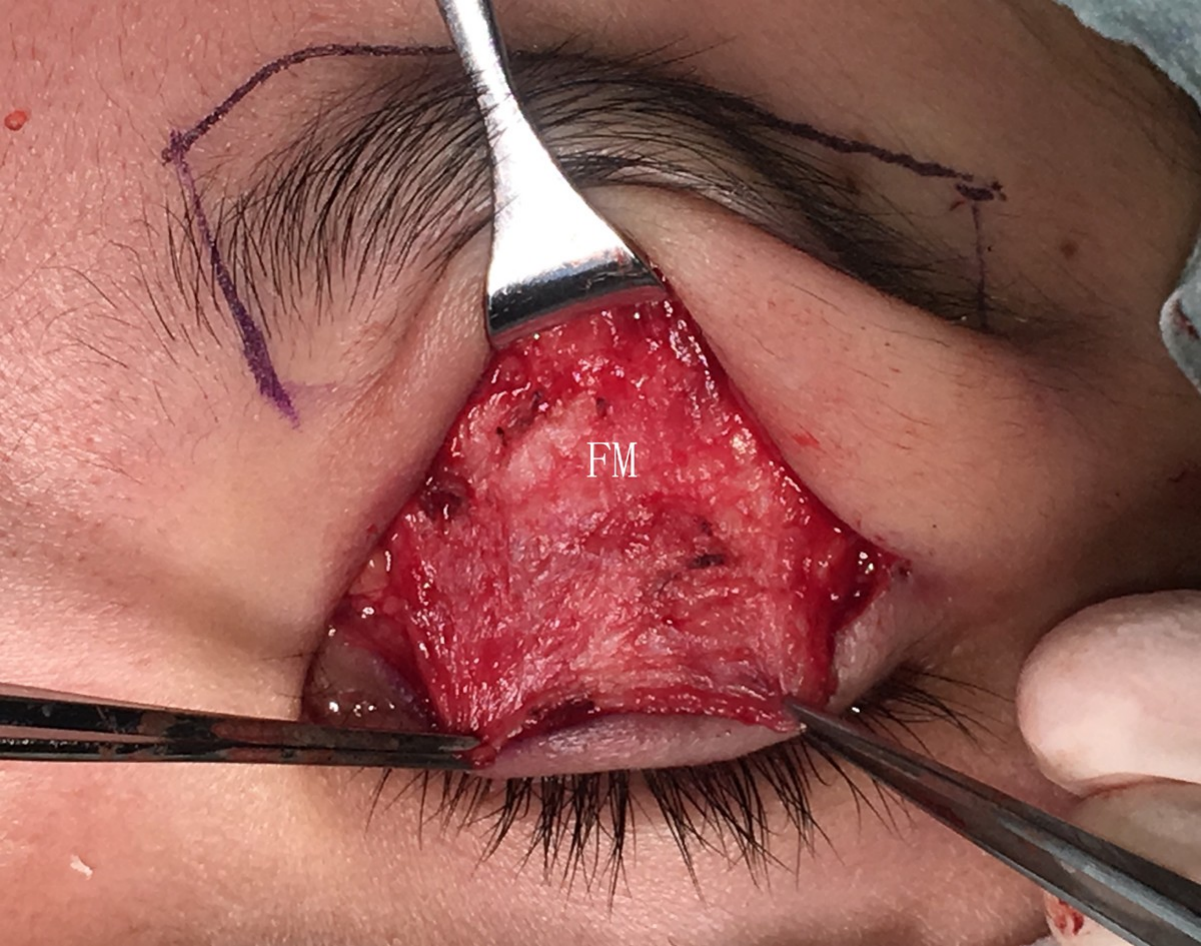
The final modified FM flap.

**Fig 8:**
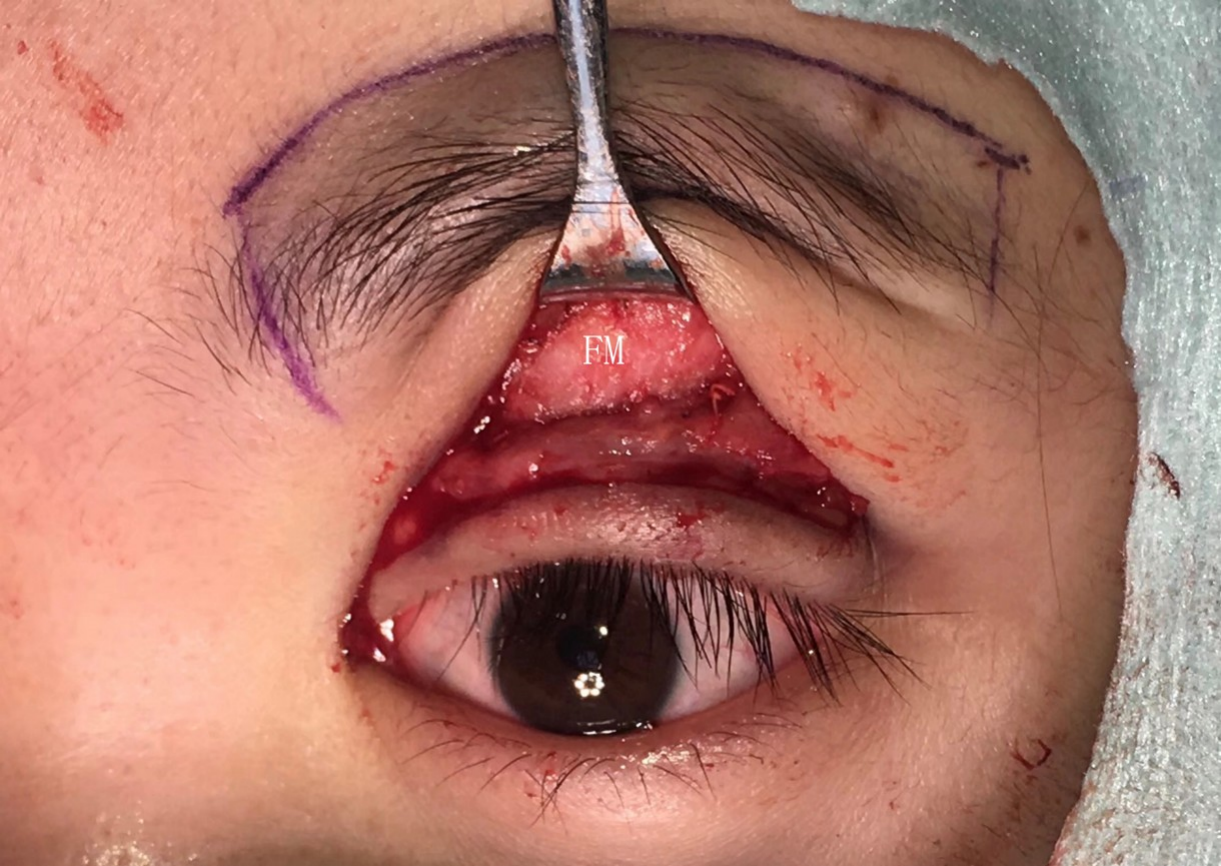
Modified FM attached to the tarsal plate with three sutures. The purple line shows the dissection boundary of the modified FM flap.

The FM flaps were different between the two groups. ① For conventional surgery, subcutaneous separation was performed in the parietal direction up to approximately 1.5 cm above the eyebrow. Two vertical incisions on the FM were made next to the supraorbital neurovascular bundle to make an FM flap with a length of approximately 1.5 cm and width of approximately 1.5 cm. A deep separation was made between the FM and the periosteum in the parietal direction up to 1–1.5 cm above the supraorbital margin (Figs 1–3). ② For the modified surgery, subcutaneous separation was performed in the parietal direction up to 0.5 cm above the eyebrow, and the medial and lateral sides of the FM needed to be subcutaneously separated to the same extent without a vertical incision (Figs 3–8). There was no deep separation above the supraorbital margin (Table 1).

**Table 1:** Conventional and modified FM flap designs.

	Conventional	Modified
**Subcutaneous separation above the eyebrow**	1.5 cm	0.5 cm
**Deep separation above the supraorbital margin**	1–1.5 cm	0
**Vertical incision**	2	None
**FM flap horizontal width**	1.5 cm	2.5 cm

The FM flap was pulled down and sutured to the upper third of the tarsal plate with three 5/0 nonabsorbable sutures. The position of the upper eyelid was adjusted to 1 to 2 mm above the healthy or expected eyelid position. When the upper eyelid position was satisfactory, the excess FM flap was removed, and the residual end of the FM flap was continuously sutured to the orbicularis oculi of the middle third of the tarsal plate using 6/0 absorbable sutures. The skin crease incision was closed with interrupted 6/0 absorbable sutures, incorporating the advanced FM flap to form the crease. At the conclusion of the surgery, a Frost suture was made in the lower eyelid to protect the cornea from exposure. A pressure bandage was applied for 24 hours.

Statistical analysis was performed using IBM SPSS Statistics 19.0. Statistical analysis of age and follow-up period between the two groups was performed with the independent samples *t*-test. A chi-squared nonparametric test was used to compare sex, laterality, undercorrection, overcorrection, angular deformity of the upper eyelid, FM paralysis, frontal hypoesthesia, severe hematoma, exposure keratitis, entropion and ectropion between the two groups.

## Results

### Baseline data

In total, 278 eyes of 200 patients with severe congenital ptosis (122 unilateral, 78 bilateral) underwent conventional or modified FM flap advancement surgery. The mean age was 7.4 (range, 2–16) years. Eighty-nine patients were female, and 111 were male. The mean preoperative MRD_1_ was -1.3 mm. Follow-up evaluations were performed from 6 months to 18 months postoperatively, with an average of 10 months. No significant difference was found between the conventional FM flap and the modified FM flap groups in terms of age, sex, the ratio of left and right affected eyes, the ratio of unilateral and bilateral affected eyes, the follow-up period or the mean preoperative MRD_1_ (p = 0.782, p = 0.670, p = 0.971, p = 0.772, p = 0.355, p = 0.350, respectively) (Table 2).

**Table 2:** Distribution of age, sex, laterality and follow-up period in the two groups.

Group	Age	Sex		Unilateral		Bilateral	Follow-up	Mean preoperative MRD_1_	Total no. of
		M	F	Right	Left		Period(months)		Patients (Eyes)
**Conventional**	7.5±5.3	57	43	37	25	38	10.3±4.1	-1.3±0.91	100 (138)
**Modified**	7.3±4.9	54	46	36	24	40	9.8±3.5	-1.4±0.87	100 (140)
***P*-value**	0.782 ^a^	0.670 ^b^		0.971 ^b^		0.772 ^b^	0.355 ^a^	0.350^a^	

^a^ based on the independent samples *t*-test.

^b^ based on the chi-squared nonparametric test.

### Success rate

**Fig 9:**
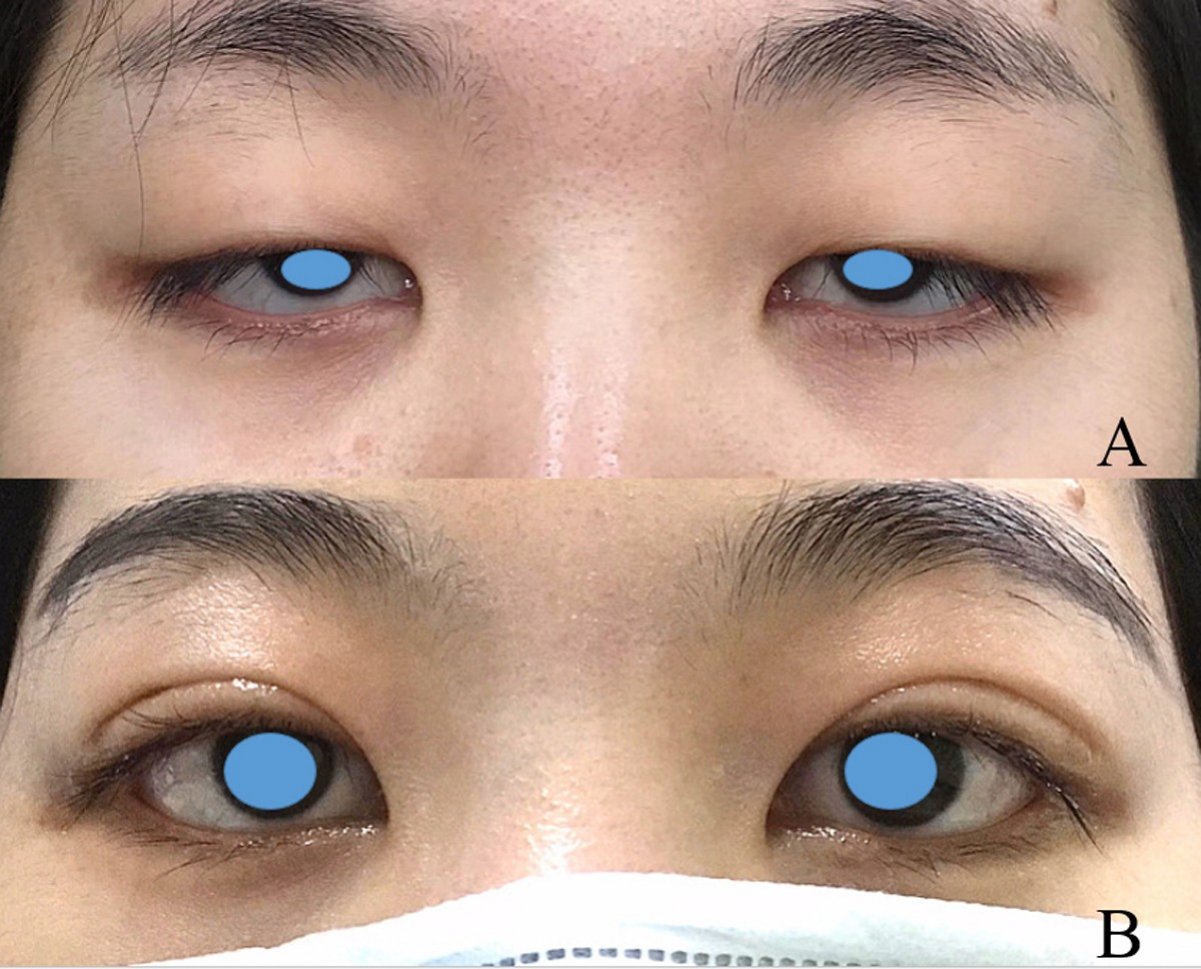
Bilateral congenital ptosis. (A) Before the modified surgery. (B) Six months after the operation.

The final success rate was 77.5% (107/138) in the conventional group and 90.0% (126/140) in the modified group (Fig 9), and the difference was statistically significant (p = 0.005). Undercorrection was observed in 31 eyes (22.5%) in the conventional group and 14 eyes (10%) in the modified group, and the difference was statistically significant (p = 0.005). There were no cases of overcorrection in either group.

### Complications

**Fig 10:**
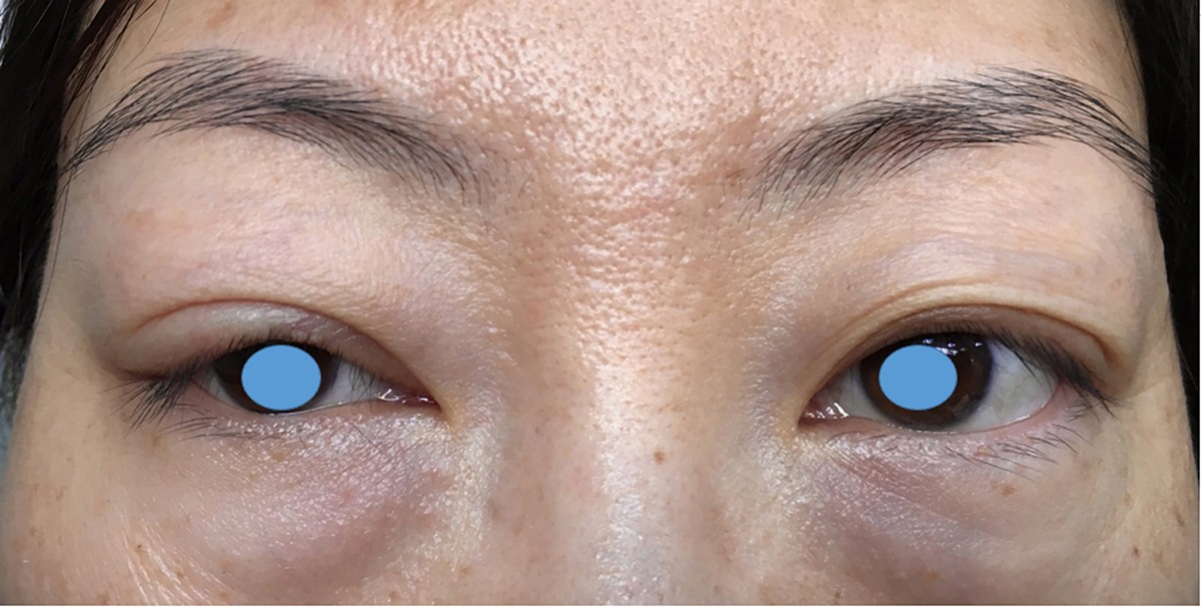
Angular deformity of the right upper eyelid that occurred six months after the conventional surgery.

In the conventional group, angular deformity of the upper eyelid was observed in 29 eyes (21.0%) (Fig 10), FM paralysis was observed in 11 (8%) eyes, frontal hypoesthesia was observed in 10 (7.2%) eyes, severe hematoma was observed in 12 (8.7%) eyes, and exposure keratitis was observed in 8 (5.8%) eyes, while in the modified group, there were no cases of angular deformity of the upper eyelid, FM paralysis, frontal hypoesthesia, severe hematoma or exposure keratitis, and the difference was statistically significant (p <0.001, p <0.001, p = 0.004, p <0.001, p = 0.011). There were no cases of entropion or ectropion in either group (Table 3).

**Table 3:** Complications in the two groups.

	Conventional group (%)	Modified group (%)	*P*-value^a^
**Undercorrection**	31 (22.5%)	14 (10.0%)	0.005
**Overcorrection**	0	0	
**Angular deformity of the upper eyelid**	29 (21.0%)	0	<0.001
**FM paralysis**	11 (8.0%)	0	<0.001
**Frontal hypoesthesia**	10 (7.2%)	0	0.004
**Severe hematoma**	12 (8.7%)	0	<0.001
**Exposure keratitis**	8 (5.8%)	0	0.011
**Entropion**	0	0	
**Ectropion**	0	0	

^a^based on the chi-squared nonparametric test.

## Discussion

The choice of surgical method to correct ptosis is decided by the levator palpebrae superioris function and the degree of ptosis. Although the degree of levator function is regarded as the most important factor, the criteria are still different for each surgeon. Payman [9] and Collin [1] insisted that levator resection could be performed in cases of levator function greater than 4 mm and that the frontal muscle should be used in cases of levator function less than 4 mm. The authors of most Chinese textbooks present the same view as Payman and Collin; that is, 4 mm is a critical value for the choice of surgical method. With severe ptosis, deeper dissections should be performed to resect more of the levator muscle but can increase the risk of injury to surrounding tissues such as the superior oblique muscle; however, with poor levator function, even if a long portion of the levator is resected, undercorrection would still occur in some cases [10].

Regarding the method of using the FM to correct severe ptosis, there are variations between those applied in China and those applied in Europe and the United States. In Europe and the United States, it is popular to use the brow suspension operation to indirectly transmit the force of the FM to the upper eyelid through various materials. In China, it is popular to use the FM flap advancement operation, which requires the creation of an FM flap and fixation of the flap to the upper tarsal plate to produce direct traction [11].

FM originates from the galea aponeurotica and ends in the subcutaneous tissue of the eyebrow skin. It is innervated by the frontotemporal branch of the facial nerve. Compared with eyebrow suspension, FM flap advancement has the following advantages: ① the frontalis muscle flap, as an autologous tissue, not only avoids the side effects of autologous fascia lata transplantation, such as muscle hernia and skin scars [12] but is also innervated and has a blood supply, which compared with that for synthetic sling materials, reduces the probability of postoperative infection, exposure or foreign body granuloma formation [13–15].② The FM is intertwined with the surrounding tissue, which can provide a stronger and more durable upper eyelid suspension than other materials. ③ With the FM flap pulled down and sutured directly to the upper tarsal plate, excessive forehead wrinkles are relieved, and FM contraction can produce a direct and effective eyelid lifting motion, which results in a more natural and dynamic eyelid shape and a more normal eyebrow location than brow suspension [11].

In 1901, Fergus was the first to report the use of FM to correct a single case of congenital ptosis [16]. His method was not well accepted until 1982, when Song described the use of the L-shaped medial FM flap to treat ptosis [17]. Since Song’s description, the application of FM flaps to treat severe ptosis has been extensively reported. There have been many variations in the shape of the FM flap, and U-shaped FM flaps appear in most Chinese textbooks in the introduction of standard FM flap advancement surgery to correct ptosis in cases of poor levator function [11].

### The conventional FM flap advancement operation has several disadvantages

① Risk of FM paralysis, frontal hypoesthesia and severe hematoma. With a vertical incision on the FM and deep dissection between the FM and periosteum, there is a risk of injury to the frontotemporal nerve and supraorbital neurovascular bundle, which may lead to FM paralysis, frontal hypoesthesia and severe hematoma. [5, 6]. In the conventional group of this study, FM paralysis occurred in 11 (8%) eyes, frontal hypoesthesia occurred in 10 (7.2%) eyes, and severe hematoma occurred in 12 (8.7%) eyes. We analyzed whether the occurrence of these complications may be related to the depth or extent of the surgical dissection or vertical incisions. The motor innervation of the FM is laterally based. Zhang reported that the mean lowest point at which the frontotemporal nerve enters the FM is 7.6 mm ±1.5 SD from the supraorbital margin [18], which indicates that there is a risk of FM denervation from vertical temporal incisions made on the FM above the supraorbital margin. In addition, the supraorbital vessels and nerve run through the frontal muscle and the galea aponeurotica into the scalp [19]. With nasal vertical incisions and deep dissection during conventional FM flap advancement, frontal hypoesthesia and severe hematoma can easily occur. ② Risk of angular deformity of the upper eyelid. In the conventional group of this study, angular deformity of the upper eyelid was observed in 29 eyes (21.0%). As the frontalis flap is fixed, it could be pulled too tightly, and the horizontal width of the conventional flap is too narrow to provide even lifting power, which may result in an unsatisfactory contour.

### Compared with the conventional FM flap advancement technique, the modified technique of this study has some advantages

① Lower risk of FM paralysis, frontal hypoesthesia and severe hematoma. The modified FM flap is made without deep dissection or vertical incisions above the supraorbital margin, which avoids the risk of injuring the frontotemporal branch of the facial nerve and the supraorbital neurovascular bundle. No complications, such as FM paralysis, frontal hypoesthesia or severe hematoma, occurred in the modified group in this study (p<0.05). ② A more natural contour of the upper eyelid. The modified FM flap in this study is wider and can cover almost the entire horizontal width of the upper eyelid, leading to a stronger and more uniform lifting power. All patients in the modified group in this study showed satisfactory postoperative eyelid contours without angulation deformity. ③ Low recurrence rate. Of the 140 eyelids treated with the modified FM flap advancement surgery, only 14 required reoperation for recurrence. This success rate (90.0%) was higher than that in the conventional group (p<0.05). The modified procedure maximizes the integrity of the FM and the connection between the FM and surrounding tissues and greatly reduces the risk of direct injury by avoiding deep separations and vertical incisions. These modifications improve the tension and stability of the FM flap, thereby reducing the recurrence rate. ④ Clear surgical field of view. The modified FM flap has a wider horizontal dimension and a shorter vertical dimension than the unmodified flap, supporting a clear field of view. ⑤ Lower incidence of exposure keratitis. All exposure keratitis cases occurred in patients with postoperative severe hematoma (p<0.05). Hematoma may cause upper eyelid retraction and increase the palpebral fissure height, which makes the cornea more exposed to the air; in addition, hematomas cause local swelling and pain, which make it difficult for children to use eye ointment to protect the cornea, leading to exposure keratitis.

There was no entropion, ectropion or overcorrection in the conventional group and the modified group, which may be due to our appropriate control of the double eyelid width design and frontal muscle tension. Epicanthus is common in Asia, so they are prone to entropion after FM flap advancement, especially on the nasal side of the upper eyelid. When the skin crease incision is designed, the width should be less than that of the tarsal plate. When the nasal skin crease incision is sutured, the deep tissue should be sutured upward to tighten the skin at the lower edge of the incision to prevent the occurrence of entropion. The movement direction of FM is basically vertical upward, which is different from that of levator muscle. If the tension of the frontal muscle flap is too high, the upper edge of the tarsal plate will be stuck at the upper edge of the orbit and bend, resulting in eyelid ectropion and overcorrection. In this case, appropriate advancement of the frontal muscle flap can effectively prevent the occurrence of the above two complications.

### Both the modified procedure and the conventional procedure have the same three disadvantages

Dry eye, lagophthalmos and slow movement of the upper eyelid during downward gaze. In addition to physical defense, the eyelid also participates in the uniform distribution of glandular secretions into the tear film to protect the eye surface from dryness. Due to the large tension, the upper eyelid movement is limited, and the palpebral fissure cannot be closed completely, which easily causes dry eyes, lagophthalmos and slow movement of the upper eyelid during downward gaze. The above shortcomings can be alleviated by compensating artificial tears in daytime and applying thick eye ointment before sleep to alleviate dry eye and avoid exposure keratitis. Movement of the head should be used as much as possible to reduce eye movement during downward gaze.

The design of this study was nonrandomized and retrospective, so the examiner and patient guardians could not be blinded, which may lead to selection bias.

In conclusion, the modified technique in this study is simple and effective in correcting severe congenital blepharoptosis. This modification not only retains the advantages of the conventional FM flap but also greatly reduces the incidence of serious complications. It makes use of clear anatomical landmarks, and the operation procedures are easily learned, both of which are especially suitable for beginners or hospitals lacking sling materials. Although the retrospective design of this study has some limitations, we believe that the modified FM flap advancement technique is worth comparing with brow suspension in future controlled trials for the treatment of severe congenital ptosis.

## Supporting information

S1 Data (XLSX)
